# Aniline-containing guests recognized by α,α’,δ,δ’-tetramethyl-cucurbit[6]uril host

**DOI:** 10.1038/srep39057

**Published:** 2016-12-13

**Authors:** Rui-Lian Lin, Guo-Sheng Fang, Wen-Qi Sun, Jing-Xin Liu

**Affiliations:** 1 College of Chemistry and Chemical Engineering, Anhui University of Technology, Maanshan 243002, China

## Abstract

The host−guest complexation of symmetrical α,α’,δ,δ’-tetramethyl-cucurbit[6]uril (TMeQ[6]) and cucurbit[7]uril (Q[7]) with a series of aniline-containing guests has been investigated by various experimental techniques including NMR, ITC, and X-ray crystallography. Experimental results indicate that both TMeQ[6] and Q[7] hosts can encapsulate aniline-containing guests to form stable inclusion complexes. However, the oval cavity of TMeQ[6] is more complementary in size and shape to the aromatic ring of the guests than the spherical cavity of Q[7]. Shielding and deshielding effects of the aromatic ring on guests lead to the remarkable chemical shifts of the TMeQ[6] host protons. The rotational restriction of the guests in the oval cavity of TMeQ[6] results in the large negative values of entropy. The X-ray crystal structure of the 1:1 inclusion complex between TMeQ[6] and *N,N*′-diethyl-benzene-1,4-diamine unambiguously reveals that the aromatic ring of the guest resides in the oval cavity of TMeQ[6].

Molecular recognition continues to be an active topic of supramolecular chemistry because of its potential applications in separation, chemical and biological sensing, drug delivery, and molecular self-assembly^1^,^2^,^3^,^4^,^5^,^6^,^7^,^8^. In general, the driving forces for molecular recognition include electrostatic interaction, hydrogen bonding, ion-dipole interaction, π–π interaction, van der Waals interaction, hydrophobicity, and frequently the combinations of them^9^,^10^,^11^,^12^,^13^,^14^,^15^. Additionally, selective molecular recognition requires size and shape complementarities of the guest to the host be taken into account. Many synthetic hosts such as cyclodextrins, calix[*n*]arenes, cyclophanes and pillar[*n*]arenes have been reported for the study of molecular recognition phenomena^16^,^17^,^18^,^19^,^20^,^21^.

Recently, there has been intensive research into the synthetic hosts, cucurbit[*n*]urils (*n* = 5–8, 10, abbreviated as Q[*n*]) and their derivatives^22^,^23^,^24^,^25^,^26^. As a class of organic cyclic oligomers, Q[*n*]s possess a hydrophobic cavity and two identical carbonyl-laced portals that can bind with all kinds of organic molecules to form inclusion complexes. For example, Q[6] has been shown to form rotaxane and pseudorotaxane constructs with a variety of alkylammonium and alkyldiammonium ions^27^,^28^,^29^,^30^,^31^,^32^. Q[7] exhibits excellent binding affinity to amantadine and ferrocene derivatives^33^. The larger Q[8] can accommodate two organic molecules simultaneously to form ternary complexes through host-stabilized charge-transfer interactions^34^. We noticed that the hydrophobic cavity of the Q[*n*]s is centrosymmetric spherical, while the encapsulated guests have a wide range of geometries, including linear, spherical, and planar.

Aniline and its derivatives are important organic compounds and widely used in the chemical industry such as dyes, rubbers, pharmaceutical preparation, plastic and paint^35^,^36^,^37^. On the other hand, aniline and its derivatives are harmful to public health and environmental quality. Therefore, how to recognize and sensing the aniline and its derivatives has become a significant environmental concern. Liu *et al*. have reported a polymeric pseudorotaxane structure, where polyaniline chain is threaded into numerous Q[7] cavities^38^. This study suggests that the Q[7] has the potential to accommodate the aniline and its derivatives. In the host-guest molecular recognition systems, we are interested in the symmetrical α,α’,δ,δ’-tetramethyl-cucurbit[6]uril^39^,^40^ (TMeQ[6], Fig. 1), a derivative of Q[6]. In contrast to the spherical cavity of above-mentioned Q[*n*]s, TMeQ[6] features an oval cavity, which enables it to display higher complementary selectivity for specific guests. From the structural point of view, the size and shape of the aromatic group in aniline and its derivatives are resemble closely to the oval cavity of TMeQ[6]. We envisioned that the TMeQ[6] has an ability to recognize and encapsulate the aniline and its derivatives.

In the present study, we investigated the host−guest complexation of TMeQ[6] and Q[7] with the positively charged aniline (**1**) and its derivatives (including *p*-methylaniline (**2**), *p-*nitrophenylamine (**3**), *p*-phenylenediamine (**4**), and *N, N*′-diethyl-benzene-1,4-diamine (**5**), Fig. 2) in aqueous solution by ^1 ^H NMR spectroscopy and isothermal titration calorimetry (ITC), and in the solid state by X-ray crystallography. It should be noted that because of the shielding and deshielding effects of the hosts, the encapsulated guest protons usually shift upfield or downfield, which is being studied intensively. However, the experimental results presented herein shown that the encapsulated guest can also generates shielding and deshielding effects, and lead to remarkable chemical shifts of the host protons. This kind of phenomenon has never been reported.

## Results and Discussion

### ^1^H NMR results on the shielding and deshielding effect of the aromatic ring

**Figure 2:**
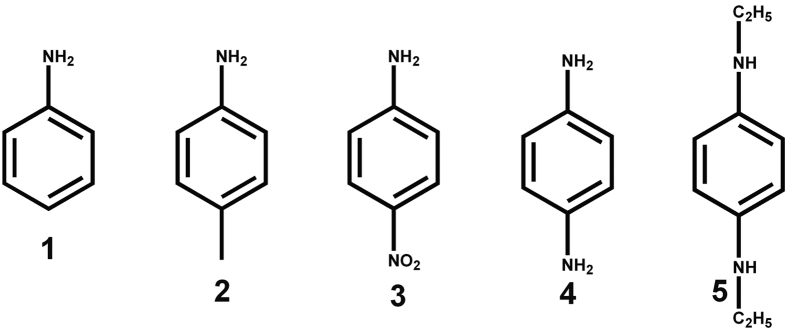
Molecular structures of guests surveyed in this work .

**Figure 3:**
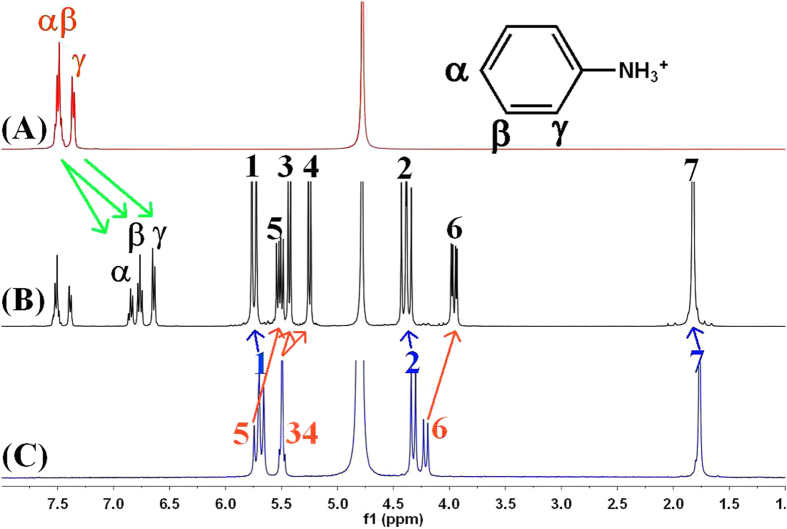
1H NMR spectra (400 MHz) of 1.3 mg guest **1**^+^ in absence (**A**) and presence of 0.62 (**B**) equiv of TMeQ[6] in 0.50 ml D_2_O at 20 °C. (**C**) shows the ^1^H NMR spectrum (400 MHz) of TMeQ[6] in 0.50 ml D_2_O at 20 °C. ^1^H NMR spectra (400 MHz) of 1.3 mg guest **1**^+^ in absence (**A**) and presence of 0.62 (**B**) equiv of TMeQ[6] in 0.50 ml D_2_O at 20 °C. (**C**) shows the ^1^H NMR spectrum (400 MHz) of TMeQ[6] in 0.50 ml D_2_O at 20 °C.

^1^H NMR experiments were used to measure the binding interaction of TMeQ[6] with the positively charged aniline and its derivatives. The ^1^H NMR spectra of **1**^+^ and **1**^+^ bound to TMeQ[6] are shown in Fig. 3. In the presence of 0.62 equiv of TMeQ[6] (Fig. 2b), the resonances for the aromatic protons (α, β and γ) shifted upfield by 0.67, 0.74 and 0.75 ppm, respectively, indicating that the guest **1**^+^ is encapsulated and form inclusion complex. When an excess amount of the guest **1**^+^ is present, the resonances of free and bound anilines were simultaneously observed in the ^1^H NMR spectra, revealing that the exchange rate between free and bound **1**^+^ is slow on the ^1^H NMR time scale. Integration of appropriate proton resonances of the TMeQ[6] host and bound **1**^+^ demonstrates a 1:1 mole ratio stoichiometric inclusion.

**Figure 4:**
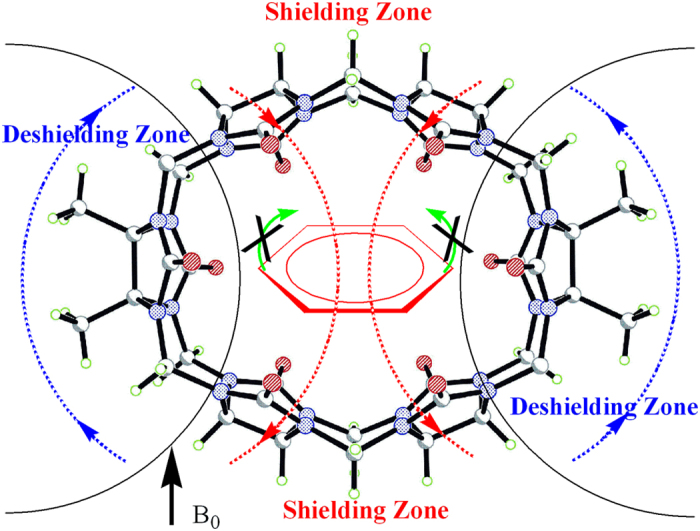
Aromatic ring-induced shielding and deshielding zones.

The most interesting feature of the binding interaction between TMeQ[6] and **1**^+^ is that the resonances of the TMeQ[6] host protons undergo remarkably chemical shifts. As can be seen in Fig. 3b, the H1, H2 and H7 resonances experienced downfield shifts of about 0.07, 0.09 and 0.06 ppm, respectively. In contrast, the resonances of H3, H4, H5 and H6 protons experienced upfield shifts of about 0.05, 0.24, 0.10 and 0.25 ppm, respectively. We ascribed this phenomenon to the shielding and deshielding effect of the aromatic ring in the guest **1**^+^. When the asymmetric **1**^+^ is encapsulated in the TMeQ[6] host, the guest **1**^+^ is impossible to rotate around the axle of the oval cavity. As a result, the aromatic ring induces two different magnetic fields (Fig. 4). The protons H3, H4, H5 and H6 are situated in the shielding zone while the protons H1, H2 and H7 are situated in the deshielding zone. At the same time, a splitting in the doublets for the TMeQ[6] host protons is also observed, reflecting two different ends of the guest **1**^+^ neared to the two portals of the TMeQ[6].

**Figure 5:**
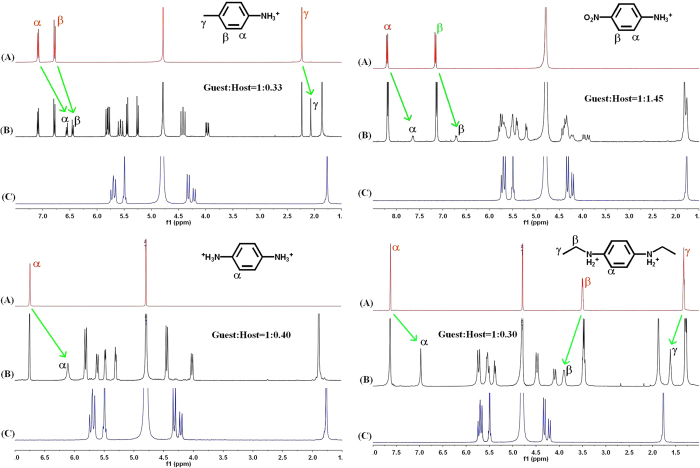
a–d. ^1^H NMR spectra (400 MHz) of guests **2**^**+**^(1.4 mg), **3**^**+**^(1.7 mg), **4**^**2+**^ (1.8 mg) and **5**^**2+**^(2.4 mg) in absence (**A**) and presence of TMeQ[6] in 0.50 ml D_2_O at 20 °C. (**C**) shows the ^1^H NMR spectrum (400 MHz) of TMeQ[6] in 0.50 ml D_2_O at 20 °C.

Similar NMR data were obtained with the other positively charged aniline-containing guests. As shown in Fig. 5, all the resonances of the aromatic protons on the aniline-containing guests experience a considerable upfield shift, suggesting that all the aromatic groups of these guests can be encapsulated into the TMeQ[6] cavity. It should be noted here that only a small portion of the guest **3**^+^ (about 10%) moved upfield from those of the free guest when more than 1.0 equiv of TMeQ[6] was added, probably because of the intense charge repulsion between the nitro-group on **3**^+^ and the carbonyl oxygens on the TMeQ[6] portals. This behavior suggests that the binding affinity of TMeQ[6] with **3**^+^ is much weaker than with other guests. In the case of guest **5**^2+^, the resonances of ethyl protons moved downfield, indicative of their positioning outside of the TMeQ[6] cavity. The downfield shift of ethyl protons may be attributed to the deshielding effect of the carbonyl-rimmed portal of TMeQ[6]. Therefore, on the basis of the ^1^H NMR characterization, the host TMeQ[6] forms 1:1 mole ratio inclusion complexes with all the positively charged aniline-containing guests.

The host–guest complexations of Q[7] with the positively charged aniline **1**^+^ and its derivatives guests (**2**^+^-**5**^2+^) are also illustrated by the ^1^H NMR spectra shown in Figure S1. The upfield shift of the aromatic protons from the original chemical shift of the free aniline-containing guests indicates the inclusion of the aromatic groups into the Q[7] cavity, generating inclusion complexes of Q[7] host with aniline-containing guests. However, no separate signals are observed for the Q[7] host protons, suggesting that the chemical environments of the Q[7] host remain unchanged after including these aniline-containing guests. It is probably that guests **1**^+^-**5**^2+^ are rotate freely around the axle of the Q[7] cavity.

### Isothermal titration calorimetry studies on the host–guest interaction

**Figure 6:**
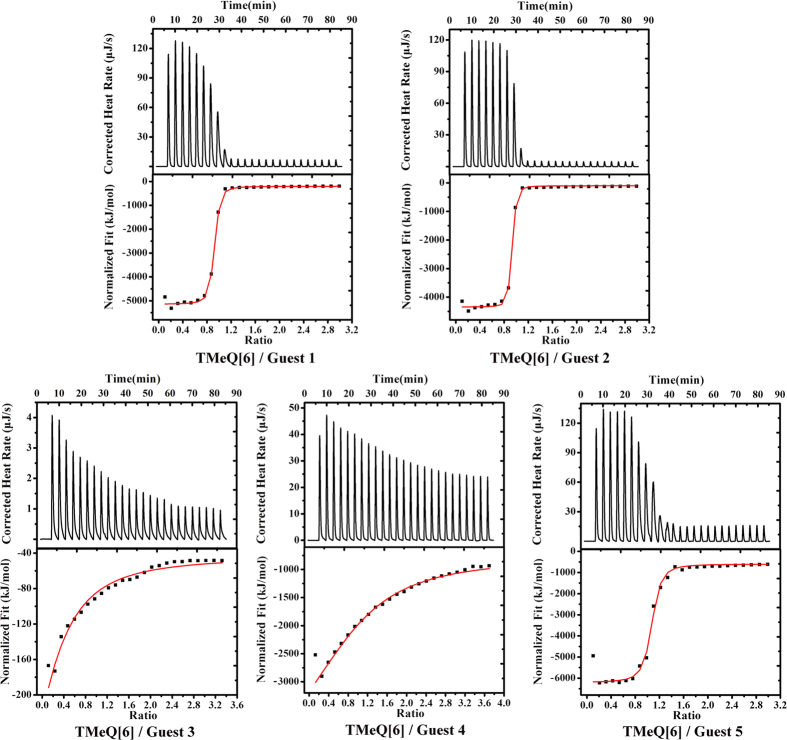
ITC profiles for the TMeQ[6] complexation with guests **1**^**+**^-**5**^2**+**^ at 298.15 K.

To determine the thermodynamic parameters and also the nature of the host–guest complexation of TMeQ[6] with aniline and its derivatives, we have used the isothermal titration calorimetry (ITC) technique, and the results are given in Figure 6 and Table 1. From the data obtained, we have found that all the enthalpy and entropy values are large negative. Obviously, the large negative value of enthalpy results from the strong host–guest interactions, including ion-dipole interactions between the positively charged nitrogens of the guest and the carbonyl oxygens on the host portal, and the van der Waals interactions between the aromatic ring of the guests and the inner wall of the TMeQ[6] host. The large negative value of entropy presumably arises from the restriction of the rotational freedom. When the aromatic ring of the guest is encapsulated within the TMeQ[6] host, the guest is restricted to rotate around axle of the oval cavity of the host. Taken together, the encapsulation of these guests within TMeQ[6] cavity is almost exclusively enthalpy driven.

The stability constants (*K*) for the complexation of the guests **2**^+^ and **4**^2+^ with TMeQ[6] are much higher than those measured for the same guests with the parent Q[6]^29^. The large *K* values change is most probably due to the enhancement of host–guest interactions, including ion-dipole and van der Waals interactions. First, the methyl groups on TMeQ[6] belong to electron donating group, indicating the carbonyl groups of TMeQ[6] have a higher dipole moment than those of the parent Q[6], which results in a stronger ion-dipole interaction. Second, the aniline-containing guests are complementary in size and shape to the oval cavity of the TMeQ[6] host, suggesting that the aniline-containing guests possess stronger van der Waals interactions with the TMeQ[6] host than with the parent Q[6].

We also carried out ITC to determine the thermodynamic parameters for the host–guest complexation of Q[7] with guests **1**^+^-**5**^2+^ (see Figure S2 and Table 1). In contrast to TMeQ[6], the host–guest complexation of Q[7] with guests **1**^+^-**5**^2+^ is driven by both enthalpy and entropy. The observed positive entropy values for the host–guest complexation of Q[7] with guests **1**^+^-**5**^2+^ further confirm that the aromatic rings of these guests in Q[7] cavity are much freer than when they were in TMeQ[6] cavity.

### Structural Analysis on the inclusion complex between TMeQ[6] and guest 5

**Figure 7:**
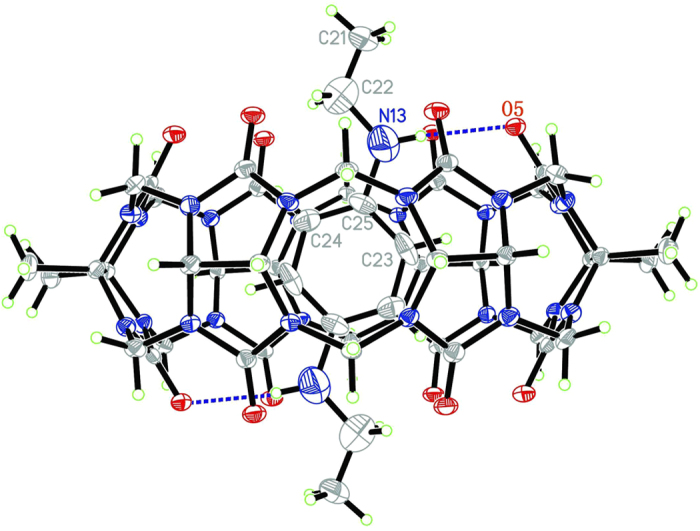
ORTEP diagrams of 5@TMeQ[6]; displacement ellipsoids are drawn at the 30% probability level. Solvate water molecules are omitted for clarity. O =** **red, C =** **grey and N =** **light blue.

To better understand the host–guest complexation between TMeQ[6] and these guests, we successfully obtained the X-ray crystal structures of the inclusion complexes *N, N*′-diethyl-benzene-1,4-diamine **5**@TMeQ[6]. The inclusion complex crystallizes with the monoclinic space group *P*2_1_/*c* and its perspective view is shown in Fig. 7. It is interesting to note that guest **5** is symmetrically threaded through the cavity of TMeQ[6]; the aromatic ring resides at the center of the cavity, whereas two terminal ethyl groups are located externally; two nitrogen atoms (N13 and N13A) of the guest **5** lie under the mean oxygen plane of the portal with 0.382 Å. This observation is in agreement with its ^1^H NMR spectroscopic data. The driving forces for such a location of the guest are likely to be hydrogen bonds between the two ammonium groups of the guest and carbonyls on two portals of the TMeQ[6] host: N13···O5 3.054(10) Å. Since the aromatic ring is complementary in size and shape to the cavity of TMeQ[6] host, the van der Waals interaction between the surfaces of the aromatic ring and the inner wall of the TMeQ[6] host also plays important roles in stabilizing the inclusion complex.

## Conclusion

In summary, we have investigated the host–guest complexation of TMeQ[6] and Q[7] with aniline and its derivatives by ^1^H NMR spectroscopy, ITC, and X-ray crystallography techniques. ^1^H NMR spectroscopy indicate that aniline-containing guests can form inclusion complexes with both TMeQ[6] and Q[7]. Because of the rotational restriction, the aromatic ring of the encapsulated guests generate interesting shielding and deshielding effects, which result in remarkable chemical shifts of the TMeQ[6] host protons. Large negative values of entropy for the host–guest complexation of TMeQ[6] with aniline-containing guests are also observed, which is mainly due to the loss of rotational freedom of the guests when they were encapsulated inside the oval cavity of the TMeQ[6] host. Compared with TMeQ[6], the Q[7] cavity is less complementary in size and shape to the aromatic ring of the aniline-containing guests, which can rotate freely in the Q[7] cavity. Present study of the molecular recognition of TMeQ[6] host to aniline-containing guests not only can be utilized in removing aniline and its derivatives from aqueous solution, but also can be exploited in the design and synthesis of (pseudo)rotaxanes, poly(pseudo)rotaxanes and catenanes.

## Experimental Section

### Materials and methods

Guests **1**–**5** were purchased from Aldrich. Guests **1**^**+**^-**5**^2**+**^were obtained by isolating the hydrochloride salts of guests **1**–**5** with acetone in water. TMeQ[6] was prepared according to a literature method^39^. All the NMR data were recorded on a Bruker DPX 400 spectrometer in D_2_O at 293.15 K.

### Single-crystal X-ray crystallography

Single crystals of inclusion complex **5**@TMeQ[6] were grown from water by solvent evaporation. Single crystal X-ray analyses for this inclusion complex were conducted at 273 K on a Bruker SMART Apex-II CCD diffractometer using graphite-monochromated Mo-*K*_α_ radiation (λ =** **0.71073 Å). Lorentz polarization and absorption corrections were applied. Structural solution and full-matrix least-squares refinement based on *F*^*2*^ were performed with the SHELXS-*97* and SHELXL-*97* program packages, respectively^42^,^43^,^44^. All non-hydrogen atoms were refined anisotropically. CCDC 1400469 contains the supplementary crystallographic data for this paper. These data can be obtained free of charge from The Cambridge Crystallographic Data Centre via www.ccdc.cam.ac.uk/ data_request/cif.

### Crystal data for 5@TMeQ[6]

[(C_8_H_16_N_2_)@(C_40_H_44_N_24_O_12_)]·16H_2_O, *M*_*r*_ =** **1505.50, Monoclinic, space group *P*2_1_/*c, a* =** **13.6298(13) Å, *b* =** **15.4295(15) Å*, c* =** **16.6342(16) Å, *β* = 112.804(3) °, *V* =** **3224.8(5) Å^3^, *Z* =** **2, *Dc* =** **1.550 g·cm^-1^, *F*(000) =** **1596, *GOF* =** **1.009, *R*_1_ =** **0.1142 (*I* > 2*σ(I*)), *wR*_*2*_ =** **0.3848 (all data).

### Preparation of the inclusion complex 5@TMeQ[6]

To a solution of TMeQ[6]·10H_2_O (61.6 mg, 0.05 mmol) in 10 ml H_2_O, guest **5** (17.6 mg, 0.10 mmol) was added. The resulting reaction mixture was stirred for 1 hour at 60 °C and filtered. Slow solvent evaporation of the filtrate in air over a period of about three weeks provided blue crystals of **5**@TMeQ[6] in 20% yield (based on TMeQ[6]). Anal. Calcd for C_50_H_92_N_26_O_28_: C, 39.89; H, 6.16; N, 24.19. Found: C, 39.71; H, 6.22; N, 24.07.

### Isothermal titration calorimetry (ITC) experiments

ITC was carried out with a Nano ITC instrument (TA, USA) at 298.15 K. All solutions were degassed prior to titration experiments. An aqueous solution (0.1 mm) of TMeQ[6] or Q[7] was placed in the sample cell (1.3 mL). A solution (1 mm) of the guest **1**^**+**^(or other guests **2**^**+**^**, 3**^**+**^**, 4**^**2+**^ and **5**^**2+**^) was added stepwise in a series of 25 injections (10 μL each). The heat of dilution was corrected by injecting the guest solution into deionized water and was subtracted. The first data point was always removed. The results were analyzed with the independent model using ORIGIN 7.0 software.

## Additional Information

**How to cite this article**: Lin, R.-L. *et al*. Aniline-containing guests recognized by α,α′,δ,δ′-tetramethyl-cucurbit[6]uril host. *Sci. Rep.* **6**, 39057; doi: 10.1038/srep39057 (2016).

**Publisher's note:** Springer Nature remains neutral with regard to jurisdictional claims in published maps and institutional affiliations.

## Supplementary Material

Supplementary Information

## Figures and Tables

**Figure 1:**
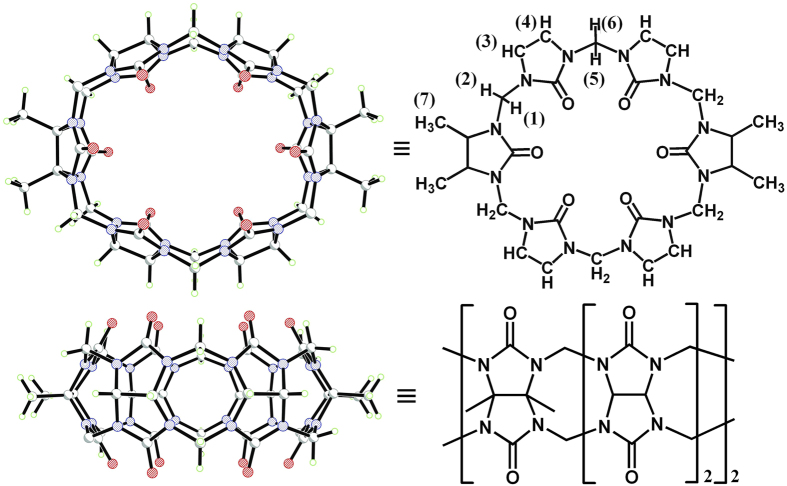
X-ray crystal structure of TMeQ[6] in top view (up) and side view (down) .

**Table 1:** The stability constants and thermodynamic parameters for the host–guest complexation of TMeQ[6] and Q[7] with aniline-containing guests.

Experiment	*K* (M^−1^)	Δ*H*° (J·mol^−1^)	*T*Δ*S*° (J·mol^−1^)
**1**^**+**^·TMeQ[6]	(6.02 ± 0.45) × 10^6^	(−4.85 ± 0.03) × 10^5^	−4.46 × 10^5^
**2**^**+**^·TMeQ[6]	(1.47 ± 0.16) × 10^7^ 8980 ± 450^a^	(−4.12 ± 0.03) × 10^5^	−3.71 × 10^5^
**3**^**+**^·TMeQ[6]	(1.69 ± 0.11) × 10^4^	(−5.72 ± 0.13) × 10^4^	−3.31 × 10^4^
**4**^**2+**^·TMeQ[6]	(1.69 ± 0.11) × 10^4^ 1860 ± 100^a^	(−3.46 ± 0.13) × 10^5^	−3.22 × 10^5^
**5**^**2+**^·TMeQ[6]	(1.96 ± 0.82) × 10^6^	(−5.61 ± 0.14) × 10^5^	−5.25 × 10^5^
**1**^**+**^·Q[7]	(4.24 ± 0.30) × 10^6^	(−3.55 ± 0.04) × 10^4^	2.29 × 10^3^
**2**^**+**^·Q[7]	(4.08 ± 0.71) × 10^6^	(−3.47 ± 0.01) × 10^4^	3.03 × 10^3^
**3**^**+**^·Q[7]	(1.37 ± 0.12) × 10^5^	(−1.30 ± 0.11) × 10^4^	1.63 × 10^4^
**4**^**2+**^·Q[7]	(5.37 ± 0.50) × 10^6^	(−3.07 ± 0.09) × 10^4^	7.73 × 10^3^
**5**^**2+**^·Q[7]	(2.68 ± 0.22) × 10^6^	(−3.29 ± 0.06) × 10^4^	3.84 × 10^3^

^a^Stability constants of Q[6] with **2**^**+**^and **4**^**2+**^were obtained from reference (41).
