# Supplementary material for: Aniline-containing guests recognized by α,α’,δ,δ’-tetramethyl-cucurbit[6]uril host
Source: Sci Rep. 2016 Dec 13;6:39057. doi: 10.1038/srep39057 (PMC5153640; doi:10.1038/srep39057)
Supplement: Supplementary Information [file srep39057-s1.doc]

**Supporting Information**

**Aniline-containing guests recognized by α,α’,δ,δ’-tetramethyl-cucurbit[6]uril host**

*_________________________________________________________________________*

*Rui-Lian Lin, Guo-Sheng Fang*, *Wen-Qi Sun, Jing-Xin Liu**,

College of Chemistry and Chemical Engineering, Anhui University of Technology, Maanshan 243002, China

jxliu411@ahut.edu.cn

**Table of Contents**

Figure S1 ^1^H NMR spectra of Q[7] with guests **1^+^**-**5**^2^**^+^**.............................................................. 1

Figure S2 ITC profiles for the Q[7] complexation with guests **1^+^**-**5**^2^**^+^** at 298.15 K.................... 2

**
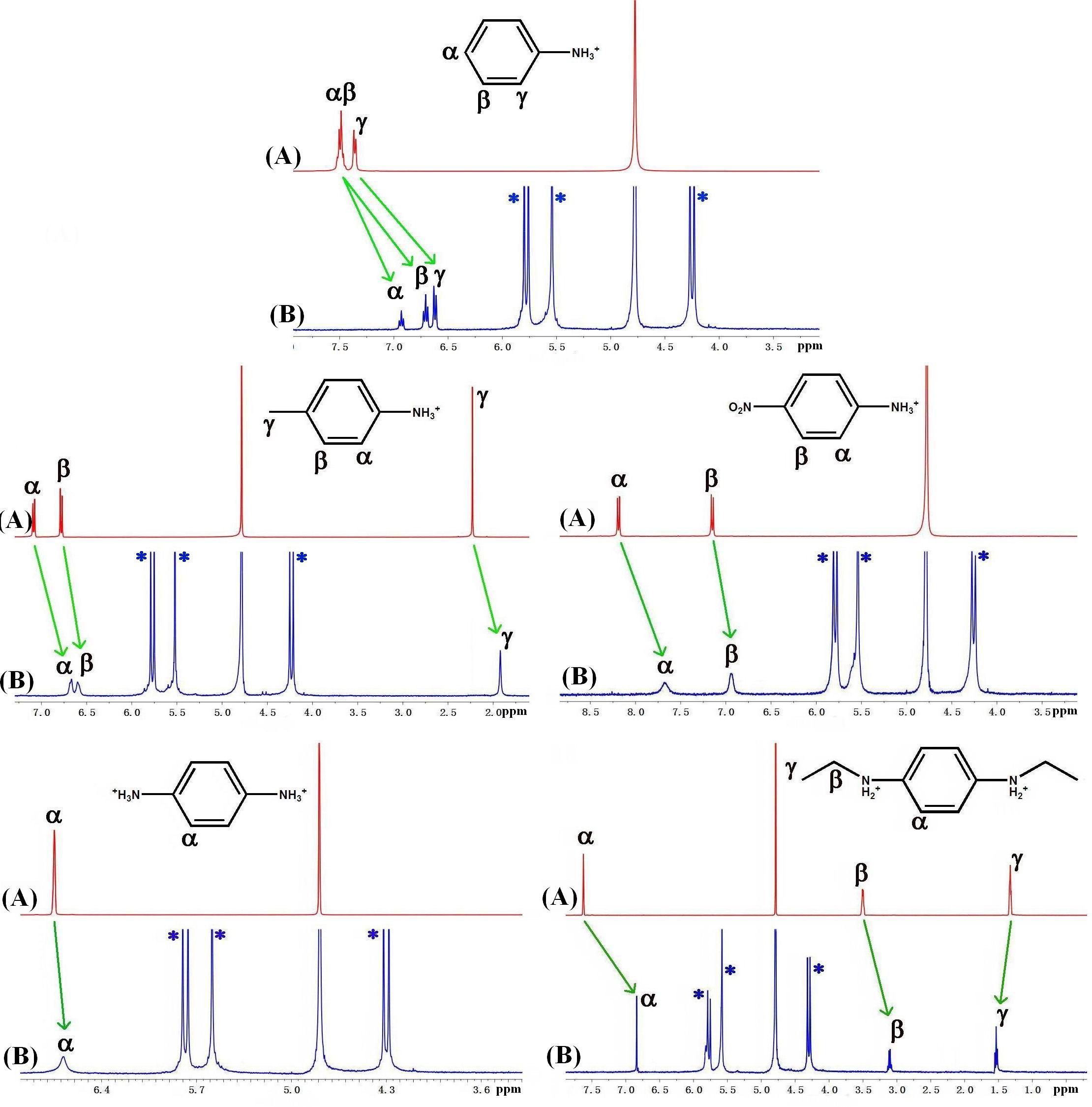
**

**Figure S1.** ^1^H NMR spectra (400 MHz) of guests **1^+^** (1.3 mg), **2^+^** (1.4 mg), **3^+^** (1.7 mg), **4^2+^** (1.8 mg) and **5^2+^** (2.4 mg) in absence (A) and presence of (B) Q[7] (*) in 0.50 ml D_2_O at 20 ºC.


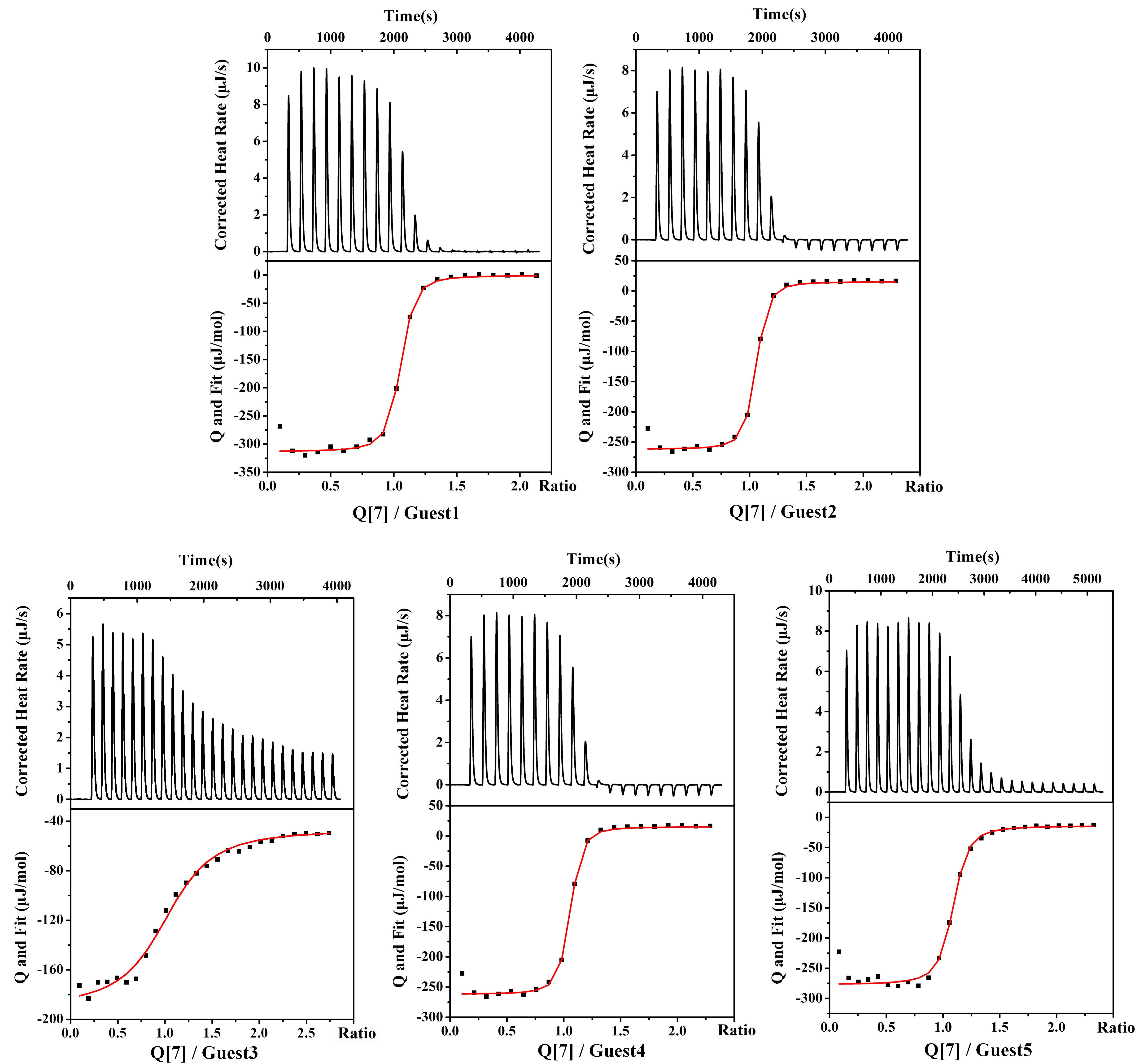
 **Figure S2.** ITC profiles for the Q[7] complexation with aniline-containing guests **1^+^**-**5**^2^**^+^** at 298.15 K.
